# What Kind of Intervention Is Effective for Improving Subjective Well-Being Among Workers? A Systematic Review and Meta-Analysis of Randomized Controlled Trials

**DOI:** 10.3389/fpsyg.2020.528656

**Published:** 2020-11-13

**Authors:** Asuka Sakuraya, Kotaro Imamura, Kazuhiro Watanabe, Yumi Asai, Emiko Ando, Hisashi Eguchi, Norimitsu Nishida, Yuka Kobayashi, Hideaki Arima, Mai Iwanaga, Yasumasa Otsuka, Natsu Sasaki, Akiomi Inoue, Reiko Inoue, Kanami Tsuno, Ayako Hino, Akihito Shimazu, Akizumi Tsutsumi, Norito Kawakami

**Affiliations:** ^1^Department of Public Health, School of Medicine, Tokyo Women's Medical University, Tokyo, Japan; ^2^Department of Mental Health, Graduate School of Medicine, The University of Tokyo, Tokyo, Japan; ^3^Center for Cancer Control and Information Services, National Cancer Center, Tokyo, Japan; ^4^Department of Public Health, Kitasato University School of Medicine, Kanagawa, Japan; ^5^Kyoto Industrial Health Association, Kyoto, Japan; ^6^Department of Psychiatric Nursing, Graduate School of Medicine, The University of Tokyo, Tokyo, Japan; ^7^Faculty of Human Sciences, University of Tsukuba, Tokyo, Japan; ^8^School of Health Innovation, Kanagawa University of Human Services, Kanagawa, Japan; ^9^Department of Mental Health, Institute of Industrial Ecological Sciences, University of Occupational and Environmental Health Japan, Fukuoka, Japan; ^10^Faculty of Policy Management, Keio University, Kanagawa, Japan

**Keywords:** subjective well-being, positive mental health, systematic review, intervention, worker, meta-analysis

## Abstract

**Objectives:** This study aimed to conduct a systematic review and meta-analysis of randomized controlled trials (RCTs) to improve subjective well-being (SWB), including evaluative, hedonic, and eudemonic well-being, and the mental component of quality of life (QOL) of working population.

**Methods:** A literature search was conducted, using PubMed, Embase, PsycINFO, and PsycARTICLES. Eligible studies included those that were RCTs of any intervention, conducted among healthy workers, measured SWB as a primary outcome, and original articles in English. Study characteristics, intervention, outcomes, and results on SWB outcomes were extracted by the investigators independently. After a brief narrative summarizing and classifying the contents of the interventions, the included outcomes were categorized into each aspect of SWB (evaluative, hedonic, and eudemonic well-being, and the mental component of QOL). Finally, the characteristics of the effective interventions for increasing each aspect were summarized, and the pooled effect of interventions on SWB was investigated by a meta-analysis. Publication bias was investigated by drawing a funnel plot and conducting Egger's test.

**Results:** From the 5,450 articles found, 39 met the inclusion criteria for the systematic review. The interventions included in this review were classified into six categories (physical activity, ergonomics, psychological, environmental, multicomponent intervention, and others). The meta-analysis from 31 studies showed that the pooled effect of included interventions on SWB was significantly positive (standardized mean difference (SMD) = 0.51; standard error (SE) = 0.10). A funnel plot showed there were extremely large or small SMDs, and Egger's test was significant. Thus, we conducted sensitivity analysis, excluding these extreme SMDs, and confirmed that the estimated pooled effect was also significantly positive. Subgroup analyses for separate types of interventions showed the effects of psychological interventions (e.g., mindfulness, cognitive behavioral based approach, and other psychological interventions) were also significantly positive.

**Conclusion:** The current study revealed the effectiveness of interventions for increasing SWB. Specifically, psychological interventions (e.g., mindfulness, cognitive behavioral based approach, and other psychological interventions) may be useful for improving SWB.

## Introduction

People's self-reports of their subjective well-being (SWB) have received attention in recent years. Subjective well-being refers to people's perceptions of their existence or their subjective view of their life experience including affective reactions as well as cognitive judgments (Diener, 1984; Russell, 2008; Martín-María et al., 2017). In recent years, Diener (2006) redefined SWB as “An umbrella term for different valuations that people make regarding their lives, the events happening to them, their bodies and minds, and the circumstances in which they live,” which has been a popular conception of SWB (Diener, 2006; Camfield and Skevington, 2008; Steel et al., 2008). Improvement of SWB is one of the major concerns for global mental health (Steptoe et al., 2015). For example, some studies reported that SWB contributes to people's lifelong health and healthy aging (Diener and Chan, 2011; Ngamaba et al., 2017). In the working population, SWB is also an important outcome associated with work-related positive outcomes such as job performance and productivity (Schulte and Vainio, 2010; Bakker and Oerlemans, 2011). Promoting positive mental health, such as SWB among workers, is an important issue in the field of occupational health research.

According to the Steptoe et al. (2015) definition, SWB has three different aspects; evaluative, hedonic, and eudemonic well-being. Evaluative well-being is evaluation of how satisfied people are with their lives, such as job satisfaction and life satisfaction. Hedonic well-being is feeling or moods such as happiness or positive affect. Also, eudemonic well-being is judgment about the meaning and purpose of life (Steptoe et al., 2015). A previous meta-analysis showed each of these aspects could have a significant protective role for mortality (Martín-María et al., 2017). In other observational studies, each of them was reported to be associated with health-related outcomes (e.g., improving cardiovascular disease, physical complaints, or depression) in the general population (Faragher et al., 2005; Wood and Joseph, 2010; De Neve et al., 2013; Lamers et al., 2015; Imamura et al., 2016) and work-related outcome (i.e., high job performance) in the working population (Bakker and Oerlemans, 2011; Christian et al., 2011; De Neve et al., 2013). Hence, enhancing each aspect of well-being—evaluative, hedonic, and eudemonic—is essential for employees' health and performance. In addition to these three aspects of SWB, some scholars have treated quality of life (QOL), especially the mental component (Medvedev and Landhuis, 2018), as a prominent aspect of SWB (Diener, 2006; Camfield and Skevington, 2008; Steel et al., 2008), while it is not included in the definition of SWB by Steptoe and colleagues (Steptoe et al., 2015). According to Skevington and Böhnke (2018), QOL is also indispensable for people's health. Thus, the mental component of QOL could also be an important aspect of SWB as well as the other three aspects of SWB.

For now, there have been an increasing number of randomized controlled trials (RCTs) to improve these aspects of SWB among healthy workers. For example, mindfulness and ergonomics interventions such as participatory training facilitated by an occupational therapist were reported to be effective in increasing evaluative well-being (e.g., job satisfaction) (King et al., 1997; Hülsheger et al., 2013). Regarding hedonic well-being, physical activity and psychological interventions such as cognitive behavioral (CB)-based approaches could improve vitality and positive affect (Atlantis et al., 2004; Unsworth and Mason, 2012). Regarding eudemonic well-being, CB-based approaches could also be effective (Bolier et al., 2014). Besides, physical activity could enhance the mental component of QOL (Atlantis et al., 2004; Brand et al., 2006). Although these RCTs have been performed, there have been few systematic reviews and meta-analyses of them, and these treated only individual aspects of SWB. For example, Knight and his colleagues conducted a systematic review of interventions to increase hedonic well-being (e.g., work engagement) (Knight et al., 2017, 2019). They reported that mindfulness could be useful (Knight et al., 2019). Next, Weiss et al. (2016) reported a meta-analysis of RCTs of behavioral intervention on eudemonic well-being (e.g., psychological well-being), although it targeted the general population. It showed that mindfulness and other psychological interventions such as cognitive behavioral therapy (CBT) could be effective. Despite these previous studies, a systematic review and meta-analysis assessing all aspects of SWB (i.e., evaluative, hedonic, eudemonic well-being, and the mental component of QOL) among workers has not been conducted. By evaluating all aspects of SWB, we could clarify the kind of intervention that would be effective for all aspects, and what kind of interventions are suitable for specific aspects. It will help us to suggest the more effective intervention for promoting workers' SWB.

This study aimed to conduct a systematic review and meta-analysis of RCTs to improve SWB, including evaluative, hedonic, and eudemonic well-being, and mental component of QOL of the working population.

## Methods

### Study Design

The present study is a systematic review and meta-analysis of RCTs that aimed to examine the intervention effect on improving SWB among workers. The Preferred Reporting Items for Systematic Reviews and Meta-Analyses (PRISMA) guidelines were followed when reporting this manuscript (Moher et al., 2009).

### Eligibility Criteria

Participants, intervention, comparisons, and outcomes (PICO) of the eligible studies were defined as follows: (P) inclusion of all healthy workers, (I) any intervention, (C) treatment as usual, and (O) SWB measured as a primary outcome. Here, we adopted the definition of evaluative, hedonic, and eudemonic well-being (Steptoe et al., 2015). Furthermore, the mental component of QOL was also included as an SWB outcome (Steel et al., 2008; Skevington and Böhnke, 2018). Here, we excluded physical and social components of QOL because they were not included in the SWB definition (Diener, 1984, 2006). In addition, eligible studies were those that were (1) RCTs, (2) written in English, and (3) original articles.

### Search and Information Sources

Search terms were preliminarily developed by an investigator (ASa) and discussed and agreed upon by all authors. The following search terms were used: (1) keywords related to SWB (e.g., satisfaction, engagement, happiness, positive emotion, purpose of life, psychological well-being, and quality of life), (2) participants (e.g., worker, employee, worksite, or workplace), and (3) study design (e.g., randomized controlled trial). The details of search terms are shown in Appendix 1, including keywords related to SWB. A systematic search was conducted in October 2016 using PubMed, Embase, PsycINFO, and PsycARTICLES.

### Study Selection

We managed all identified studies within a Microsoft® Excel (Washington, USA) file. After excluding duplicated records (by ASa), the remaining articles were shared by 10 investigators (ASa, KI, KW, YA, EA, HE, NN, YK, HA, and MI), and pairs of them independently assessed the title and abstract of each article to identify eligible studies according to the eligibility criteria (sifting phase). At this phase, we excluded studies that clearly did not meet the criteria and included the others (studies that met the criteria and those we could not assess the criteria according to the title and abstract) in a full-text review. In the next phase, pairs of investigators independently reviewed the full texts that were included as eligible studies. During the full-text review, when the investigators disagreed on the eligibility, the disagreements were solved by consensus of all authors. The reasons why studies were excluded were recorded at the full-text review phase.

### Data Collection Process and Data Items

Ten investigators (ASa, KI, KW, YA, EA, HE, NN, YK, HA, and MI) extracted information from each of the included studies for a systematic review. The year of publication, country the study was conducted, characteristics of the participants, intervention, condition of the control group, and outcomes, and result on SWB outcomes were extracted. After extraction, all authors confirmed the collected information to reach consensus in this process.

For the meta-analysis, means and standard deviations (SDs) of SWB at baseline and post-intervention surveys, and the number of participants at analyses of intervention and control groups were collected. Pairs of four investigators (ASa, KI, KW, NS) independently collected this information from each study. We contacted the corresponding authors of any studies that did not report this information or contained unclear information.

### Risk of Bias in Individual Studies

Six investigators (ASa, KI, KW, HE, AI, and NS) independently assessed the included study quality using the risk of bias assessment tool of the Grading of Recommendation Assessment, Development, and Evaluation (GRADE) system (Higgins, 2011), which evaluates randomized controlled study based on nine items: (1) random sequence generation, (2) allocation concealment, (3) blinding of participants and personnel, (4) blinding of providers, (5) blinding of outcome assessment, (6) blinding of data analysis, (7) incomplete outcome data, (8) selective reporting, and (9) other bias (e.g., cross over bias). Each item was then graded as high, unclear, or low. Discrepancy among the six investigators was settled by discussion among all authors.

### Synthesis of Results and Meta-Analysis

First, a brief narrative summarizing and classifying the contents of the interventions was written. Next, the included outcomes were categorized into each aspect of SWB (evaluative, hedonic, and eudemonic well-being, and the mental component of QOL). Finally, the characteristics of the effective interventions for increasing each aspect were summarized. This process was conducted mainly by the first author, and the synthesis of results was confirmed by all authors.

Because some studies reported multiple effect sizes of SWB on the same construct (Puig-Ribera et al., 2008; Strijk et al., 2013), it was inappropriate to independently treat each effect size when calculating the pooled effect size (Cheung, 2014; Assink and Wibbelink, 2016). Thus, for the analysis to estimate the pooled effect of interventions on SWB, three-level random-effects meta-analysis was conducted (Cheung, 2014; Assink and Wibbelink, 2016). First, standardized mean differences (SMDs) of SWB between the intervention and control groups and standard errors (SEs) for each combination of a study and an outcome were calculated. Next, we conducted three-level random-effects meta-analysis by using R with the rma.mv function of the metafor package (Cheung, 2014; Assink and Wibbelink, 2016). We assessed the heterogeneity by using the Q statistic (Assink and Wibbelink, 2016). Publication bias was investigated by drawing a funnel plot and conducting Egger's test, where, for simplicity, we did not consider the data structure having multiple effect sizes from the same studies. Based on the funnel plot, a sensitivity analysis was conducted for studies that reported relevant SMDs or their standard errors (SEs). Subgroup meta-analyses were also conducted separately for types of interventions and each aspect of SWB.

## Results

### Study Selection

**Figure 1:**
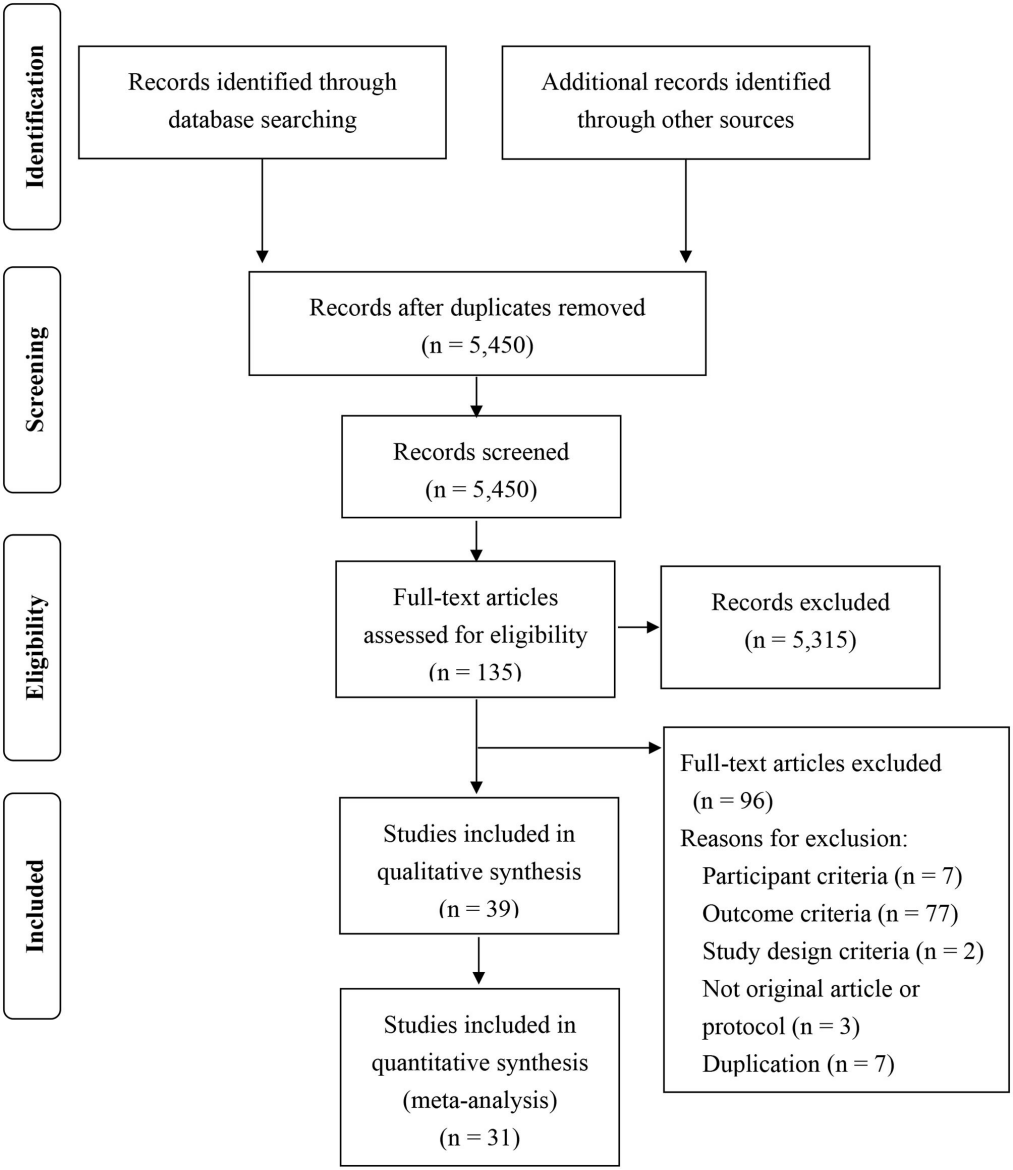
Flow diagram.

Our initial search of four databases revealed 5,474 articles overall. After removing duplicates, 5,450 articles were included in the sifting phase. Next, 5,315 articles were excluded, and 135 articles proceeded to full-text review. Following this process, 39 studies were included in the qualitative review (Figure 1). For the meta-analysis, 31 studies were used. From eight of 39 studies or their authors (King et al., 1997; Bittman et al., 2003; Schrijnemaekers et al., 2003; Sjogren et al., 2006; Haukka et al., 2010; Backman et al., 2011; Figl-Hertlein et al., 2014; Linzer et al., 2015), sufficient data were not available to calculate SMDs with SEs.

### Study Characteristics

The characteristics of 39 studies are shown in Table 1. The interventions included in this review were classified into six categories (physical activity intervention, ergonomics intervention, psychological intervention, environmental intervention, multicomponent intervention, and others). Seven of the included studies were classified as physical activity intervention (Atlantis et al., 2004; Brand et al., 2006; Sjogren et al., 2006; Puig-Ribera et al., 2008; Hartfiel et al., 2011; Strijk et al., 2013; Mansi et al., 2015); three were ergonomics intervention (King et al., 1997; Haukka et al., 2010; Figl-Hertlein et al., 2014); 21 were psychological intervention, including mindfulness (Hülsheger et al., 2013; Aikens et al., 2014; Shonin et al., 2014; Van Berkel et al., 2014; Allexandre et al., 2016; Crain et al., 2017), CB-based approach (Bond and Bunce, 2000; Billings et al., 2008; Sanders et al., 2011; Unsworth and Mason, 2012; Vuori et al., 2012; Bolier et al., 2014; Umanodan et al., 2014; Barbosa et al., 2015), and other psychological interventions (Waite and Richardson, 2004; Fillion et al., 2009; Feicht et al., 2013; Coffeng et al., 2014; Tuckey and Scott, 2014; Morgan and Harris, 2015; Muller et al., 2016); three were environmental intervention (Linzer et al., 2015; Stansfeld et al., 2015; Alhassan et al., 2016), two were multicomponent intervention (Russell, 2008; Sforzo et al., 2012); and three were others (Bittman et al., 2003; Schrijnemaekers et al., 2003; Backman et al., 2011).

**Table 1:** The characteristics of the studies included in the systematic reviews (*N* = 39).

**Author/Year**	**Country**	**Population**	**Gender Number (%) of men**	**Age Mean (SD)**	**Core intervention component**	**SWB outcomes (scale if applicable)**	**Result on SWB outcomes**
**PHYSICAL ACTIVITY**							
Puig-Ribera et al., 2008	Spain	Employees of university	All: 21 (30.0%)	Not listed	Walking program Period: 9 weeks Number and hours of session: not listed Instrument: pedometer and a map with some examples of walks Provider: not listed	Mental health (SF-12): Mental component of QOL Vitality (SF-12): Hedonic	Mental health: 0 Vitality: 0
Mansi et al., 2015	New Zealand	Employees of large meat processing plant	Int: 40 (12.2%) Cont: 14 (48.4%)	Int: 43 (14.9) Cont: 40 (12.2)	Walking program Period: 12 weeks Number and hours of session: not listed Instrument: physical activity booklet Provider: psychotherapist	Mental health (SF-36): Mental component of QOL	Mental health: 0
Sjogren et al., 2006	Finland	Employees of the city of Kuopio central administration	All: 24 (26.7%)	All: 45.7 (8.6)	Light resistance training, guidance on postural and movement control Period: 15 weeks Number and hours of session: not listed Instrument: resistance equipment Provider: psychotherapist	Life satisfaction (the scale from Ojanen, 1994 and 2000): Evaluative Meaning of life (the scale from Ojanen, 1994 and 2000): Eudemonic	Life satisfaction: 0 Meaning of life: 0
Brand et al., 2006	Germany	Employees (office and blue color workers)	Int: 36 (69.2%) Cont: 47 (90.4%)	Percent per age groups; age 20–35, 10.9%; age 36–45, 50.0 %; age 46–55, 28.2 %; age 56–65, 10.9%	Muscular relaxation, strengthening, coordination and flexibility exercises Period: 13 weeks Number and hours of session: not listed Instrument: not listed Provider: fitness coach	Psychological domain of quality of life (the World Health Organization Quality of Life inventory): Mental component of QOL Job satisfaction (the Life Satisfaction Questionnaire): Evaluative	Psychological domain of quality of life: + job satisfaction: 0
Hartfiel et al., 2011	England	University employees	Int: 3 (15.0%) Cont: 1 (5.0%)	Int: 40.6 (11.4) Cont: 38 (9.58)	Dru yoga intervention Period: 6 weeks Number and hours of session: 60 min class per week Instrument: CD and home practice form Provider: certified instructor	Life purpose and satisfaction (the Inventory of Positive Psychological Attitudes): Eudemonic	Life purpose and satisfaction: +
Atlantis et al., 2004	Australia	Star City casino employees	Int: 9 (45.0%) Cont: 11 (45.8%)	Int: 30 (6.8) Cont: 33 (8.3)	Aerobic and weight-training exercise Period: 24 weeks Number and hours of session: not listed Instrument: personalized e-mail Provider: not listed	Vitality (SF-36): Hedonic Mental health (SF-36): Mental component of QOL	Vitality: + Mental health: +
Strijk et al., 2013	Netherlands	Employees from academic hospital	Int: 93 (25.3%) Cont: 86 (23.7%)	Int: 52.5 (4.8) Cont: 52.3 (4.9)	A vitality exercise program (VEP); providing free fruit, personal coaching, yoga group, and aerobic session Period: 6 months Number and hours of session: not listed Instrument: not listed Provider: qualified instructor	Vitality (RAND-36 vitality scale): Hedonic Vitality (UWES): Hedonic	Vitality (RAND-36 vitality scale): 0 Vitality (UWES): 0
**ERGONOMICS**							
Figl-Hertlein et al., 2014	Australia	Teachers of secondary school	Not listed	Not listed	Ergonomics individual training (exercise and functional training), and stress management training Period: 5 months Number and hours of session: 2 sessions (3–4 h) Instrument: not listed Provider: licensed psychotherapist	Mental health (SF-36): Mental component of QOL Emotional well-being (AVEM): Hedonic	Mental health: 0 Emotional well-being: 0
Haukka et al., 2010	Finland	Kitchens of schools, kindergartens and nursing homes	Int: 167 (63.5%)Cont: 143 (59.3%)	Int: Range = 19–63 Median = 46 Cont: Range = 19–62 Median = 47	Ergonomics participatory training Period: approximately 11–14 months Number and hours of session: 8 sessions (total 28 h, each 3–5 h) Instrument: not listed Provider: researcher	Job satisfaction (“How satisfied are you with your present work?”): Evaluative	Job satisfaction: –
King et al., 1997	Not listed	Employees of manufacturing industry	Not listed	Not listed	Ergonomics participatory training with job redesign Period: 2–5 weeks Number and hours of session: 2 sessions (1 h) Instrument: not listed Provider: researcher, occupational therapist and safety professional	Job satisfaction (The Minnesota Satisfaction Questionnaire): Evaluative	Job satisfaction: +
**PSYCHOLOGY (MINDFULNESS)**							
Hülsheger et al., 2013	Germany	Employees in hospitals, schools, kindergartens, and medical practices	All: 18 (28.1%)	All: 38.6 (11.1)	Mindfulness-based cognitive therapy and mindfulness-based stress reduction (MBSR), self-training Period: 2 weeks (10 working days) Number and hours of session: no session Instrument: diary booklet, a CD, postcard, and daily e-mail Provider: not listed	Job satisfaction [five items from Judge, Locke, Durham, and Kluger (1998)]: Evaluative	Job satisfaction: +
Crain et al., 2017	Canada and United States	Teacher	11% Number of men is not listed	46.9 (9.2)	Mindfulness training program based on MBSR, group session and homework Period: 8 weeks Number and hours of session: 11 group sessions (2–7 h/sessions, total 36 h) Instrument: not listed Provider: instructors having formal professional training of MBSR	Satisfaction with work life (“Overall, how satisfied are you with your present teaching job?”): Evaluative Satisfaction with home life (“Overall, how satisfied are you with your life at home?”): Evaluative	Job satisfaction: + Life satisfaction: +
Van Berkel et al., 2014	Netherlands	Employees from Dutch research	Int: 36.4% Cont: 28.9% Number of men is not listed	Int: 46.0 (9.4) Cont: 45.1 (9.6)	Mindfulness-based training, free , lunch walking, and buddy-system, group session and e-coaching Period: 6 months Number and hours of session: 8 weekly group sessions (90 min) Instrument: e-coaching, CD, booklet Provider: certificated trainer	Work engagement (UWES): Hedonic	Work engagement: 0
Aikens et al., 2014	Michigan	Dow employees	Not listed	Range = 18–65	Mindfulness program, group session and individual online training Period: 7 weeks Number and hours of session: 7 times weekly (1 h) Instrument: web site and workbook Provider: certified medicine physician with MBSR training	Vigor (Shirom Vigor Scale): Hedonic	Vigor: +
Allexandre et al., 2016	United States	Employees of call center	All: 16.8% Number of men is not listed	All: 40.0 (12.6)	Online mindfulness stress management program (WSM), with weekly group meeting (WSMg1), with weekly group meeting and expert clinical support (WSMg2) Period: 8 weeks Number and hours of session: 8 weekly group sessions (1 h) Instrument: online program, CD, and diary article Provider: licensed clinical counselor and social worker	Mental health (SF-36): Mental component of QOL Vitality (SF-36): Hedonic	Mental health: + (WSM and WSMg1) Vitality: + (WSM and WSMg1)
Shonin et al., 2014	United Kingdom	Employees with middle management responsibility	Int: 56.9% Cont: 56.9% Number of men is not listed	Int: 40.14 (8.11) Cont: 39.91 (8.67)	Meditation Awareness Training (MAT), group and individual session Period: 8 weeks Number and hours of session: 8 weekly group sessions (90 min), and 4 weekly individual sessions (50 min) Instrument: CD Provider: researcher having psychotherapy and meditation teaching experience	Job satisfaction (Abridged Job in General Scale): Evaluative	Job satisfaction: +
**PSYCHOLOGY (cb BASED APPROACH: CBT)**							
Umanodan et al., 2014	Japan	Employees in a manufacturing company	Int: 135 (95.1%) Cont: 109 (90.1%)	Int: 39.7 Cont: 38.0 SD is not listed	Computer based stress management (problem-solving, time management, assertion and delegation, cognitive reconstruction and causal attribution), individual training Period: 7.4 weeks Number and hours of session: 6 sessions Instrument: e-mail Provider: the author	Job satisfaction (BJSQ): Evaluative Work engagement (UWES-J): Hedonic	Job satisfaction: 0 Work engagement: 0
**Author/year**	**Country**	**Population**	**Gender Number (%) of men**	**Age Mean (SD)**	**Core intervention component**	**SWB outcomes (scale if applicable)**	**Result on SWB outcomes**
Bond and Bunce, 2000	Not listed	People in a large media organization	All: 15 (50.0 %)	36.43 (9.72)	Acceptance commitment therapy (ACT) training, group session Period: 3 months Number and hours of session: 3 sessions (3.25 h per session) Instrument: not listed Provider: not listed	Intrinsic job satisfaction (work and life attitude survey): Evaluative	Intrinsic job satisfaction: 0
Billings et al., 2008	United States	Employees in a major technology company	All: 91 (29.4%)	Percent per age groups; age 20–29, 24.4%; age 30–39, 51.1%; age 40–49, 20.2%; age 50–59, 3.3%; age 60–69, 1.0%	Online stress and mood management training (goal setting, problem solving, and cognitive reconstruction) Period: 3 months Number and hours of session: not listed Instrument: not listed Provider: not listed	Positive mood (the Positive and Negative Affect Schedule): Hedonic	Positive mood: 0
**PSYCHOLOGY (cb BASED APPROACH: CBT**)							
Bolier et al., 2014	Netherlands	Nurses and allied health professionals	Int: 43 (22.9%) Cont: 31 (17.4%)	Int: 42 (11.4) Cont: 38 (12.1)	Tailored online interventions based on CBT Period: about 4 weeks−5 months Number and hours of session: a few sessions or modules Instrument: web site and e-mail Provider: not listed	Positive mental health (The Mental Health Continuum — Short Form): Eudemonic	Positive mental health: +
**PSYCHOLOGY (cb BASED APPROACH: COGNITIVE APPROACH)**							
Unsworth and Mason, 2012	Not listed	White-collar professional technical staff in the public sector	Int: 22 (57.9%) Cont: 20 (60.6%)	Int: 46.78 (range = 37–59) Cont: 44.65 (range = 24–58) SD in not listed	Online self-leadership training (self-management strategies and cognitive restructuring) Period: 10 weeks Number and hours of session: 5 modules (2 h), 1 module per 2 weeks Instrument: not listed Provider: an expert facilitator	Positive affect (Job Affect Scale): Hedonic	Positive affect: +
Sanders et al., 2011	Australia	Employees in various organization (having a child aged between 1 and 16 years)	The majority of parents participating in the study were mothers (72.4%)	Not listed	Workplace Triple P: training of work-family balance coping skills (e.g., cognitive reconstruction), and positive parenting skills, group sessions and telephone consultations Period: 8 weeks Number and hours of session: 4 times group session (2 h), and 4 individual telephone consultations (15 to 30 min) Instrument: workbook Provider: trained practitioner	Job satisfaction (Work and Life Attitude Scale): Evaluative	Job satisfaction: +
**PSYCHOLOGY (CB BASED APPROACH: BEHAVIORAL APPROACH)**							
Vuori et al., 2012	Finland	Employees in human resources development departments and occupational health services	Int: 50 (13.6%) Cont: 36 (10.3%)	Int: 50.47 (6.49) Cont: 49.67 (6.44)	The enhancement of career management skills (e.g., communication and assertion), group session Period: 3–7 days Number and hours of session: 5 sessions (4 h), or over 3 full days Instrument: not listed Provider: trainer	Work engagement (UWES-9): Hedonic	Work engagement: 0
Barbosa et al., 2015	Portugal	Workers in aged care facilities	All participants were women	Int: 43.37 (10.00) Cont: 45.90 (8.04)	Person centered care (PCC) based psycho-educational (PE) intervention (e.g., time management and problem-solving), group session Period: 8 weeks Number and hours of session: 8 weekly sessions (90 min) Instrument: hand-outs Provider: psychotherapist	Job satisfaction (the short-form Minnesota Satisfaction Questionnaire): Evaluative	Job satisfaction: 0
**PSYCHOLOGY (OTHERS)**							
Waite and Richardson, 2004	United States	Managers and employees in large government organization	Int: 12 (16.4%) Cont: 12 (15.6%)	Percent per age groups; age 18–33, 59.3%; age 34–49, 28.0%; over 50; 12.0%	Resiliency training program, group session Period: 5 weeks Number and hours of session: 5 weekly sessions (7 h), and follow-up review session for managers were provided every other week (1–2 h) over 6 weeks Instrument: not listed Provider: trainer	Purpose in life (the Purpose in Life Test): Eudemonic Job satisfaction (the SURVEY2000 IRS/NTEU Employee Satisfaction instrument): Evaluative	Purpose in life: + Job Satisfaction: +
**Author/year**	**Country**	**Population**	**Gender Number (%) of men**	**Age Mean (SD)**	**Core intervention component**	**SWB outcomes (scale if applicable)**	**Result on SWB outcomes**
Fillion et al., 2009	Canada	Palliative care nurses	Int: 1.8% Cont: 0% Number of men is not listed	Int: 44.96 (9.61) Cont: 43.13 (11.56)	Meaning centered training; covering five principal themes of Viktor Frankl's logotherapy, group session Period: 4 weeks Number and hours of session: 4 weekly sessions Instrument: facilitator manual book Provider: facilitator licensed psychologist and received training	Job satisfaction (General Satisfaction subscale of the Job Diagnostic Survey): Evaluative The spiritual quality of life (the Spirituality subscale of the Functional Assessment of Chronic Illness Therapy): Mental component of QOL The emotional quality of life [the Vigor/Activity subscale of the Shortened Profile of Mood States (POMS-37)]: Hedonic	Job satisfaction: 0 The spiritual quality of life: 0 The emotional quality of life: 0
Morgan and Harris, 2015	England	Workers in a medium-sized, further education college (during a period of organizational downsizing)	22 (33.3%)	45.18 (8.33)	The work-related self-affirming implementation intention (WS-AII) Period: not listed (one time session) Number and hours of session: one time Instrument: not listed Provider: not listed	Job satisfaction (the 16-item job satisfaction scale): Evaluative	Job satisfaction: 0
Muller et al., 2016	Germany	Nurses in community hospital	Int: 5.6% Cont: 5.9% Number of men is not listed	Int: 44.67 (9.34) Cont: 42.74 (9.91)	Selection, Optimization, Compensation (SOC) training; training of coping with job demand or job resource, group session Period: 9 months Number and hours of session: 6 sessions (0.5–8 h, interval: 2–8 weeks) Instrument: manuals, worksheets, and diary Provider: trainer (experienced occupational health professional)	Mental well-being (WHO-5): Hedonic	Mental well-being: +
Feicht et al., 2013	Germany	Employees in local insurance company	Int: 13 (24.1%) Cont: 18 (38.3%)	Int: 37.61 (7.72) Cont: 36.77 (10.42)	Online happiness training (e.g., “How do you feel? Check your state of mind”) Period: 7 weeks Number and hours of session: 7 weekly sessions (10–15 min) Instrument: e-mail Provider: not listed	Happiness and satisfaction (Visual Analog Scale): Hedonic Mental well-being (WHO-5): Hedonic	Happiness and satisfaction: + Mental well-being: +
Tuckey and Scott, 2014	Australia	Fire-fighters after potentially traumatic events (PTE)	All: 61 (91%)	Not listed	Critical incident stress debriefing (CISD), group session Period: one session Number and hours of session: one session (90 min) Instrument: not listed Provider: trained and experienced mental health professionals and peer supporters	Quality of life (Quality of life enjoyment and satisfaction questionnaire-short form): Mental component of QOL	Quality of life: 0
Coffeng et al., 2014	Netherlands	Office employees of a financial service provider	Int: 73 (61.9%) Cont: 65 (61.3%)	Int: 43.6 (10.3) Cont: 40.7 (9.2)	The social environmental intervention consisted of group motivational interviewing (GMI) Period: 6 weeks Number and hours of session: 3 times (90 min) Instrument: not listed Provider: trained team leader	Work engagement (UWES): Hedonic	Work engagement: 0
**ENVIRONMENT**							
Linzer et al., 2015	New York	Primary care clinician	Int: 39 (46.9 %) Cont: 41 (49.4 %)	Int: 48.3 (8.9) Cont: 46.4 (9.4)	Each clinic chooses a variety of methods to improve work life (e.g., improving communication and workflow) Period: not listed Number and hours of session: not listed Instrument: not listed Provider: not listed	Job satisfaction (Physician job satisfaction scale): Evaluative	Job satisfaction: +
Alhassan et al., 2016	Ghana	Staffs in health facilities accredited by the National Health Insurance Authority (NHIA).	Int: 40% Cont: 30%	Int: 38.3 (14.4) Cont: 36.5 (13.4)	Systematic Community Engagement (SCE) Intervention, assessing and improving of health service quality (e.g., staff attitude) Period: about 1 year Number and hours of session: not decided (on a regular basis for one year) Instrument: not listed Provider: trained facilitator	Staff motivation (Staff were asked to rank their motivation levels on 19 workplace motivation proxies): Evaluative	Staff motivation: +
Stansfeld et al., 2015	Not listed	Employees and managers in National Health Service (NHS) Mental Health Trust	Int: 74 (26.2%) Cont: 10 (15.0%)	Aged over 50 Int: 21 (31%) Cont: 112 (40%)	E-learning program for managers based on the Health and Safety Executive (HSE) management standards for work-related stress, face to face session and support by telephone Period: 3 months Number and hours of session: 1–2 modules weekly Instrument: not listed Provider: trained facilitator	Employee well-being (the Warwick Edinburgh Mental Wellbeing Scale): Hedonic	Employee well-being: 0
**MULTI COMPONENT**							
Roussel et al., 2015	Not listed	Hospital employees with an increased risk for the development of low back pain	Int: 5 (16.1%) Cont: 7 (18.4%)	Int: 41.4 Cont: 40.4 SD in not listed	A multidisciplinary prevention program for low back pain (LBP): physical activity, ergonomics, and psychological training Period: 3 months Number and hours of session: 10 group sessions (1 h), and 5 individual sessions Instrument: not listed Provider: physiotherapists, dietician, and occupational therapists	Vitality (SF-36): Hedonic Mental health (SF-36): Mental component of QOL	Vitality: 0 Mental health: 0
**Author/year**	**Country**	**Population**	**Gender Number (%) of men**	**Age Mean (SD)**	**Core intervention component**	**SWB outcomes (scale if applicable)**	**Result on SWB outcomes**
Sforzo et al., 2012	New York	Employees in the company's New York City main branch where more than 11,000 were employed	44 (45.8%)	34.5 (7.48)	The multipoint educational intervention: physical activity and psychological (stress management) intervention Period: 12 weeks Number and hours of session: several times educational sessions and weekly text messages, and twice cafeteria tours Instrument: web site, the fitness facility, and discount for healthy meal choices in the cafeteria Provider: not listed	Life satisfaction (The five-item Satisfaction with Life Scale): Evaluative Job satisfaction (Michigan Organizational Assessment Questionnaire Job Satisfaction Subscale): Evaluative	Life satisfaction: 0 Job satisfaction: 0
**OTHERS**							
Schrijnemaekers et al., 2003	Netherlands	Caregivers for elderly persons	Int: 7 (4.6%) Cont: 10 (7.2%)	Int: 35.2 (9.3) Cont: 37.7 (8.6)	Emotion-oriented care training for caregivers (e.g., learning non-verbal communication toward the resident), group session, homework Period: 8 months Number and hours of session: 2 clinical lessons (1 h), 6-day training program, and 3 supervision meetings Instrument: video Provider: the qualified and experienced teacher of a professional training organization	Job satisfaction of the professional caregivers (Maastricht Work Satisfaction Scale for Healthcare): Evaluative	Satisfaction with opportunities for self-actualization: + Satisfaction with head of the ward: + Satisfaction with quality of care: 0 Satisfaction with contact with colleagues: 0 Satisfaction with contact with residents: 0
Backman et al., 2011	United States	Low-wage workers of apparel manufacturers or food processors	Int: 135 (34.5%) Cont: 46 (33.6%)	Int: 32.6 (8.3) Cont: 33.9 (10.1)	Providing fresh fruit at workplace Period: 12 weeks Number and hours of session: 3 days a week Instrument: fruit delivery Provider: fruit delivery company	Job satisfaction (using 3 items, including workers' satisfaction with their jobs, supervisors/managers, and companies): Evaluative	Job satisfaction: 0
Bittman et al., 2003	United States	Employees in a non-profit continuing care retirement community	24 (21.4%)	45.3 SD in not listed	Recreational music making intervention (e.g., a mind-body wellness exercise, activity using shaker, and playing drum), group session Period: 6 weeks Number and hours of session: 6 sessions (1 h) Instrument: hand drums, sound shapes, auxiliary percussion instruments , and a clavi nova Provider: trained facilitator	Vigor/activity (POMS): Hedonic	Vigor/activity: +

+, favorable effect; –, unfavorable effect; 0, no effect; SWB, subjective well-being; Int, intervention; Cont, control.

### Physical Activity Intervention

The interventions aiming to promote participants' physical activity were classified as this category. Among seven studies, two were walking interventions (Puig-Ribera et al., 2008; Mansi et al., 2015), two were yoga interventions (Hartfiel et al., 2011; Strijk et al., 2013), and three were other physical exercise interventions such as light resistance training (Sjogren et al., 2006), strengthening (Brand et al., 2006), and aerobic and weight-training exercise (Atlantis et al., 2004). The exercises such as muscular relaxation or strengthening improved the mental component of QOL (Brand et al., 2006); yoga improved eudemonic well-being (e.g., life purpose and satisfaction) (Hartfiel et al., 2011); and aerobic and weight-training improved hedonic well-being (e.g., vitality) and the mental component of QOL (Atlantis et al., 2004).

### Ergonomics Intervention

The interventions using ergonomic approaches were classified as this category. Among three studies, one was individual-based ergonomic training such as functional training of postural mechanics: sitting, standing, and walking (Figl-Hertlein et al., 2014), and two were both individual and environment-based training, such as participatory ergonomic training or implementing ergonomic job redesign changes (King et al., 1997; Haukka et al., 2010). One study reported that participatory training had significantly unfavorable effects on evaluative well-being (e.g., job satisfaction) (Haukka et al., 2010). However, the other study reported a favorable effect on it (King et al., 1997). Therefore, the results of the effect on evaluative well-being (e.g., job satisfaction) were inconsistent.

### Psychological Intervention

We classified interventions using any psychological approach as this category. Psychological interventions were divided into three categories including mindfulness, CB-based approach, and other psychological interventions.

### Mindfulness

Six mindfulness intervention studies were included. Among these, three were mindfulness-related group sessions (Aikens et al., 2014; Van Berkel et al., 2014; Crain et al., 2017), one was self-training (Crain et al., 2017), and the other was a web-based program (Allexandre et al., 2016). In addition, one meditation awareness training (MAT) intervention was also reported (Shonin et al., 2014). These mindfulness programs were effective for improving evaluative well-being (e.g., job satisfaction and life satisfaction) (Hülsheger et al., 2013; Shonin et al., 2014; Crain et al., 2017), hedonic well-being (e.g., vigor/vitality) (Aikens et al., 2014; Allexandre et al., 2016), and the mental component of QOL (Allexandre et al., 2016).

### Cognitive Behavioral-Based Approach

We classified the interventions using either cognitive approach (e.g., cognitive reconstruction, causal attribution, among others) or behavioral approach (e.g., problem-solving, assertiveness training, among others) or both as this category. Among eight CB-based approaches, four were CBT (Bond and Bunce, 2000; Billings et al., 2008; Bolier et al., 2014; Umanodan et al., 2014), two were cognitive approach (Sanders et al., 2011; Unsworth and Mason, 2012), and two were behavioral approach (Vuori et al., 2012; Barbosa et al., 2015). Of four CBT studies, three were computer- or online-based CBT interventions (Billings et al., 2008; Bolier et al., 2014; Umanodan et al., 2014), and one was group training of ACT (Bond and Bunce, 2000). Both of the studies applying a cognitive approach used the cognitive reconstruction technique (Sanders et al., 2011; Unsworth and Mason, 2012). Of those, one adopted an online intervention (Unsworth and Mason, 2012), and the other adopted a group session style (Sanders et al., 2011). Both of the studies applying a behavioral approach used the assertiveness training and problem-solving training by group session style (Vuori et al., 2012; Barbosa et al., 2015). Of these CB-based approaches, online-based CBT improved eudemonic well-being (positive mental health) (Bolier et al., 2014), and two behavioral approaches also increased hedonic well-being (e.g., positive affect) (Unsworth and Mason, 2012) and evaluative well-being (e.g., job satisfaction) (Sanders et al., 2011).

### Other Psychological Interventions

Seven studies were classified as psychological interventions other than mindfulness or CB-based approaches. Resiliency training (Waite and Richardson, 2004), meaning-centered training for nurses (Fillion et al., 2009), the work-related self-affirming implementation intention (Morgan and Harris, 2015), selection, optimization, Compensation (SOC) training (group session aiming to coping with job demand or job resource) (Muller et al., 2016), online happiness training (Feicht et al., 2013), critical incident stress debriefing (Tuckey and Scott, 2014), and group motivational interviewing (Coffeng et al., 2014) were included. Of these, resiliency training improved eudemonic well-being (e.g., purpose in life) and evaluative well-being (e.g., job satisfaction) (Waite and Richardson, 2004), SOC training increased hedonic well-being (e.g., mental well-being) (Muller et al., 2016), and online happiness training increased hedonic well-being (e.g., happiness, satisfaction, and mental well-being) (Feicht et al., 2013).

### Environmental Intervention

The interventions approaching environmental factors were classified as this category. Three studies adopted environmental interventions. For example, improvement of workplace environment such as communication or workflow (Linzer et al., 2015), conducting discussions among staff, clients, or residents for improving health service quality (Alhassan et al., 2016), and manager training (e.g., how to support employees with problem) (Stansfeld et al., 2015) were conducted. Of these, improvement of workplace environment increased evaluative well-being (e.g., job satisfaction) (Linzer et al., 2015), and improving health service quality increased also evaluative well-being (e.g., staff motivation) (Alhassan et al., 2016).

### Multicomponent Intervention

We classified interventions using any combination of the above interventions (physical activity, ergonomics, psychological, and environmental intervention) in this category. Two studies were multicomponent educational interventions including physical activity, ergonomics, and psychological components (Sforzo et al., 2012; Roussel et al., 2015). Neither of them had a significant effect on SWB outcomes.

### Other Interventions

The interventions that did not fit any category were classified as other interventions. Emotion-oriented care training for caregivers (Schrijnemaekers et al., 2003), providing fresh fruit at the workplace (Backman et al., 2011), and recreational music making intervention (Bittman et al., 2003) were included. Emotion-oriented care training increased evaluative well-being (e.g., job satisfaction) (Schrijnemaekers et al., 2003), and recreational music making intervention improved hedonic well-being (e.g., vigor/activity) (Bittman et al., 2003).

### The Characteristics of the Effective Interventions for Improving Each Aspect of SWB

Based on the aspects of SWB, we classified included studies into evaluative, hedonic, eudemonic well-being, and mental component of QOL. About half out of all included studies measured evaluative (18 studies) or hedonic well-being (19 studies). On the other hand, studies assessing eudemonic well-being and the mental component of QOL were fewer (four and eight studies, respectively).

### The Effective Interventions for Improving Evaluative Well-Being

As to evaluative well-being, job satisfaction, and life satisfaction were used as outcome measures. Psychological interventions including mindfulness (Hülsheger et al., 2013; Shonin et al., 2014; Crain et al., 2017), cognitive approaches (Sanders et al., 2011), resiliency training (Waite and Richardson, 2004), ergonomics (King et al., 1997), environmental interventions (Linzer et al., 2015; Alhassan et al., 2016), and other interventions (e.g., emotion-oriented care training for care givers) (Schrijnemaekers et al., 2003) were reported as significantly effective interventions for improving evaluative well-being.

### The Effective Interventions for Improving Hedonic Well-Being

Outcome measures of hedonic well-being, happiness, emotional or mental well-being, vigor/vitality, work engagement, and positive affect were reported. Physical activity (aerobic and weight training) (Atlantis et al., 2004), psychological interventions, such as mindfulness (Aikens et al., 2014; Allexandre et al., 2016), cognitive approaches (Unsworth and Mason, 2012), happiness training (Feicht et al., 2013), and SOC training (Muller et al., 2016), and other interventions (relational music making) (Bittman et al., 2003) were reported as useful interventions to increase hedonic well-being.

### The Effective Interventions for Improving Eudemonic Well-Being

Positive mental health and purpose or meaning of life were used as outcome measure of eudemonic well-being. Physical activity (yoga) (Hartfiel et al., 2011), psychological interventions such as CBT (Bolier et al., 2014), and resiliency training (Waite and Richardson, 2004) were reported as effective approaches for increasing eudemonic well-being.

### The Effective Interventions for Improving the Mental Component of Quality of Life

The mental component of SF-36, SF-12, or other scales of QOL was used as outcome measures. Physical activity (e.g., coordination and flexibility exercise) (Brand et al., 2006), aerobic and weight training (Atlantis et al., 2004), was reported as effective approaches for improving the mental component of QOL.

### Risk of Bias Within Studies

Table 2 shows the summary of risk of bias assessed by using the GRADE system (Higgins, 2011). For almost all studies, the items of blinding of participants and personnel, providers, and outcome assessment, were graded as high risk. On the other hand, all studies showed low risk of random sequence generation.

**Table 2:** Analysis by grading of recommendation assessment, development, and evaluation (GRADE) risk of bias tool.

	**Random sequence generation**	**Allocation concealment**	**Blinding of participants and personnel**	**Blinding of providers**	**Blinding of outcome assessment**	**Blinding of data analysis**	**Incomplete outcome data**	**Selective reporting**	**Other bias**
Puig-Ribera et al., 2008	+	?	-	?	-	?	?	?	+
Mansi et al., 2015	+	+	-	-	-	?	+	+	+
Sjogren et al., 2006	+	+	-	-	-	?	+	?	-
Brand et al., 2006	+	+	-	-	-	?	-	?	-
Hartfiel et al., 2011	+	?	-	-	-	?	?	?	-
Atlantis et al., 2004	+	-	-	-	-	?	+	?	+
Strijk et al., 2013	+	+	-	-	-	+	+	+	+
King et al., 1997	+	?	-	-	-	?	?	?	+
Haukka et al., 2010	+	+	-	-	-	?	+	?	-
Figl-Hertlein et al., 2014	+	+	-	-	-	?	?	+	-
Hülsheger et al., 2013	+	?	-	?	-	?	?	?	?
Crain et al., 2017	+	?	-	-	-	?	?	?	-
Van Berkel et al., 2014	+	?	-	-	-	-	+	-	-
Aikens et al., 2014	+	?	-	-	-	?	+	?	-
Allexandre et al., 2016	+	?	-	-	-	?	+	?	+
Shonin et al., 2014	+	+	-	-	-	?	+	?	+
Umanodan et al., 2014	+	?	-	-	-	?	+	?	+
Bond and Bunce, 2000	+	?	-	-	-	?	?	?	+
Billings et al., 2008	+	?	-	-	-	?	?	?	+
Bolier et al., 2014	+	+	-	-	-	?	+	-	-
Unsworth and Mason, 2012	+	?	-	-	-	?	?	?	+
Sanders et al., 2011	+	?	-	-	-	?	?	?	+
Vuori et al., 2012	+	+	-	-	-	?	?	?	+
Waite and Richardson, 2004	+	?	?	?	?	?	?	?	+
Fillion et al., 2009	+	?	-	-	-	?	?	?	?
Morgan and Harris, 2015	+	?	-	-	-	?	+	?	+
Muller et al., 2016	+	?	?	-	-	+	?	?	?
Feicht et al., 2013	+	?	-	-	-	?	+	?	+
Tuckey and Scott, 2014	+	+	-	-	-	?	-	?	+
Linzer et al., 2015	+	?	-	-	-	?	?	?	-
Alhassan et al., 2016	+	?	-	-	-	?	+	-	+
Stansfeld et al., 2015	+	?	-	-	-	?	?	-	-
Roussel et al., 2015	+	?	-	-	?	?	+	?	-
Sforzo et al., 2012	+	?	-	-	-	?	+	-	+
Coffeng et al., 2014	+	?	-	-	-	?	+	?	+
Barbosa et al., 2015	+	?	-	-	-	?	?	?	+
Schrijnemaekers et al., 2003	+	?	-	-	-	?	+	?	?
Backman et al., 2011	+	?	-	-	-	?	?	?	-
Bittman et al., 2003	+	?	-	-	-	?	?	?	-

*+, low risk of bias, ?, unclear risk of bias, –, high risk of bias*.

### Meta-Analysis

**Figure 2:**
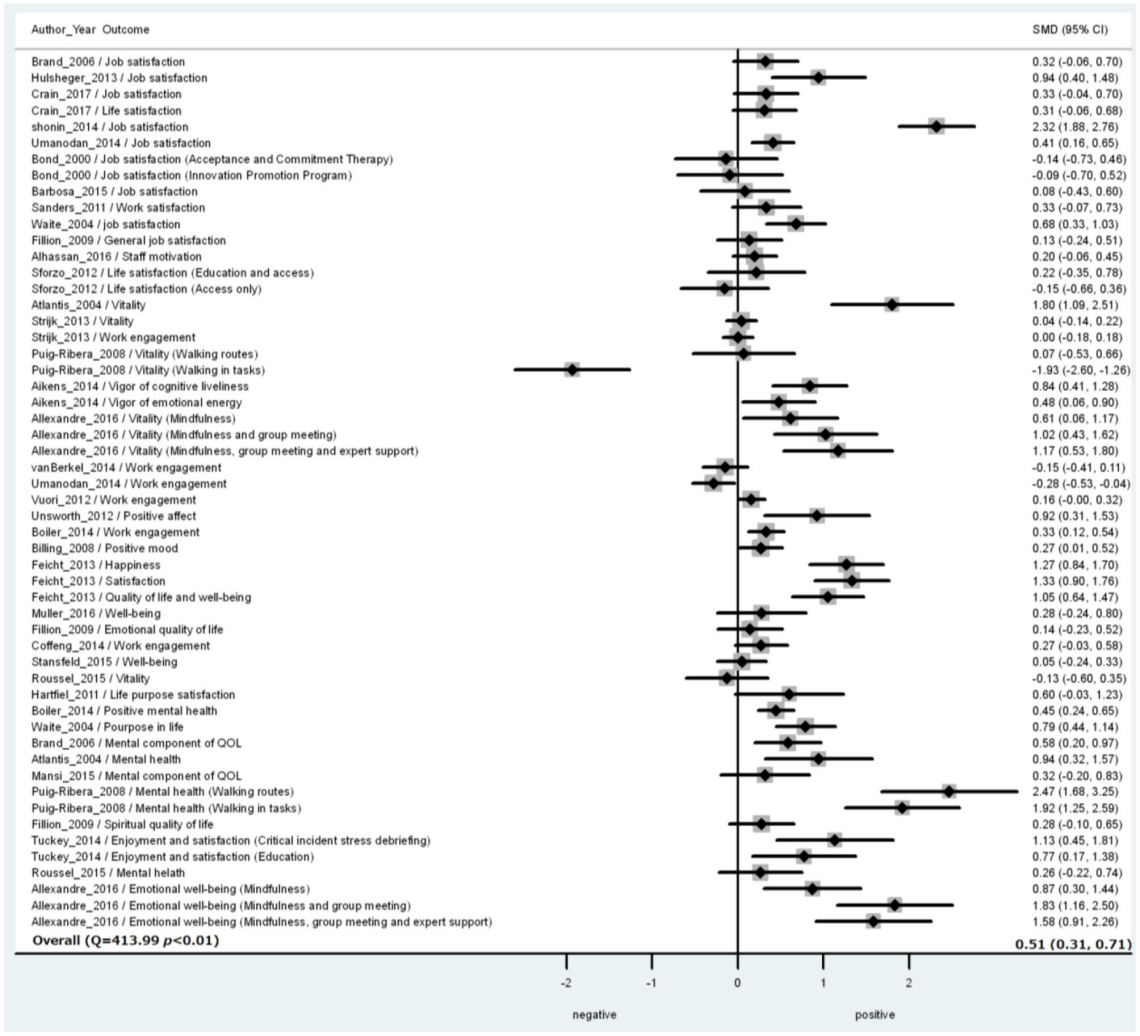
Forest plot of intervention effects [standard mean differences (SMD)] on subjective well-being (SWB) among 31 studies. Each study may include multiple outcomes. SMD and 95% CIs for an individual study were calculated based on a combination of study and outcome, where a positive effect means favorable results for an intervention group compared to a control group. The overall effect was estimated by using a three-level random-effect model considering multiple outcomes nested in the same studies. The heterogeneity was tested by Q statistic.

**Figure 3:**
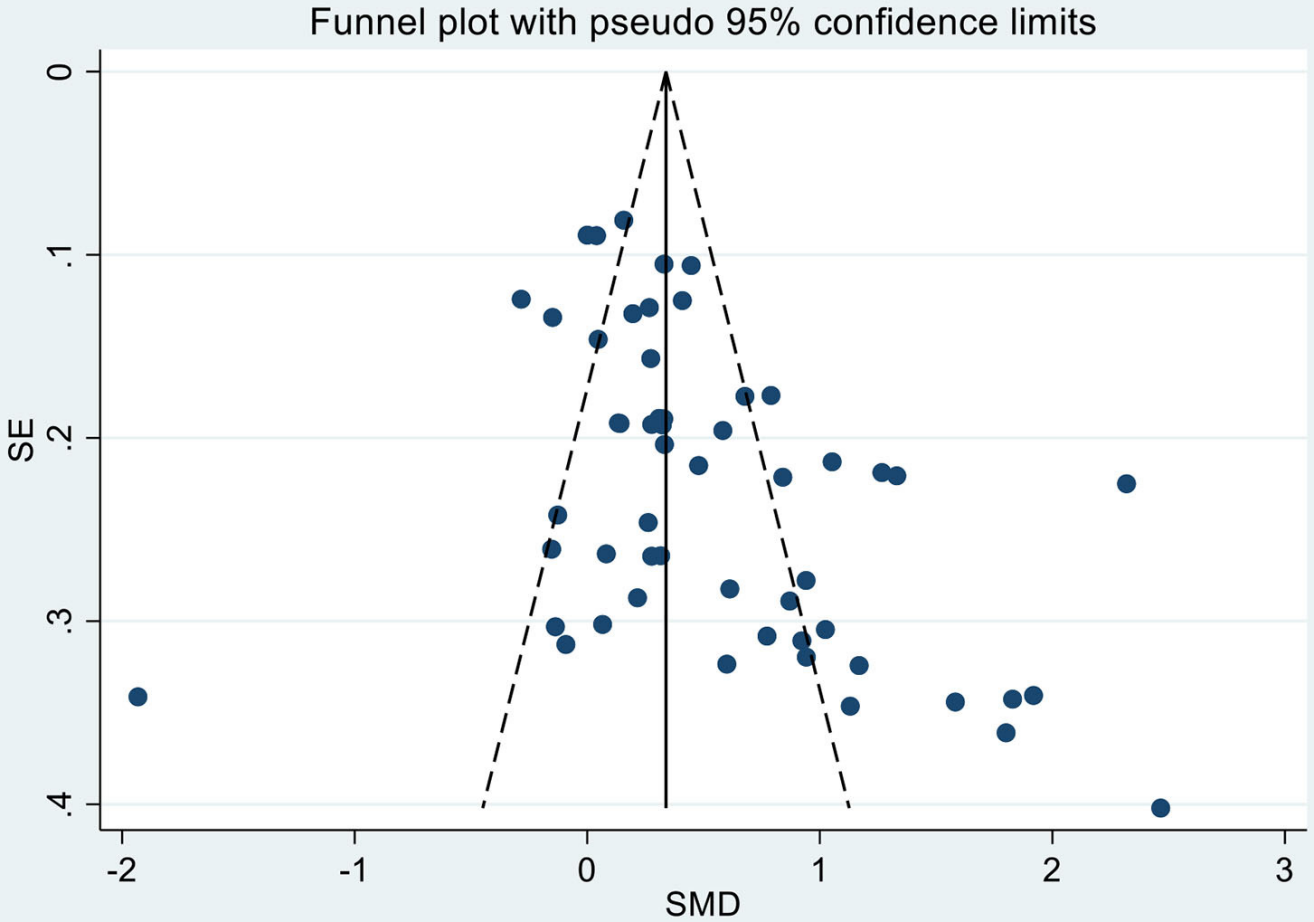
Funnel plot for standardized mean differences (SMDs) of subjective well-being (SWB) and standard errors (SEs) for 31 studies.

The result of the three-level random-effects meta-analysis of 54 SMDs from 31 studies is shown in Table 3 and Figure 2. The pooled effect of included interventions on SWB was significantly positive (SMD = 0.51; SE = 0.10; *p* < 0.01) (Table 3 and Figure 2). The heterogeneity was statistically significant (Q = 413.99, *p* < 0.01) (Table 3). Among these SMDs, 16 were extremely large or small; Egger's test was significant (*p* < 0.001, Figure 3). We conducted the sensitivity analysis for 38 SMDs, excluding these 16 extremely large or small SMDs (Atlantis et al., 2004; Puig-Ribera et al., 2008; Unsworth and Mason, 2012; Feicht et al., 2013; Hülsheger et al., 2013; Shonin et al., 2014; Tuckey and Scott, 2014; Allexandre et al., 2016). The estimated pooled effect on SWB based on 38 SMDs from 26 studies was significantly positive (SMD = 0.26; SE = 0.05; *p* < 0.01), with a significant heterogeneity (Q = 94.665, *p* < 0.01). The result of Egger's test was not significant (*p* = 0.07).

**Table 3:** The pooled effects of intervention on subjective well-being (SWB) and test of heterogeneity: three-level random-effects meta-analysis.

	**Significance test(s) of SMD** **=** **0**				**Tests of heterogeneity**		
	**SMD**	**SE**	***t***	***P***	Heterogeneity statistic (Q)*	**Degrees of freedom**	***p***
Overall	0.51	0.10	5.22	<0.01	413.99	53	<0.01
**BY INTERVENTION**							
Physical activity	0.58	0.32	1.81	0.10	135.72	11	<0.01
Mindfulness	0.86	0.34	2.54	0.03	122.88	12	<0.01
CB based approach	0.22	0.09	2.60	0.03	33.32	10	<0.01
Other psychological	0.56	0.16	3.46	0.01	49.03	11	<0.01
Environmental	0.13	0.10	1.32	0.41	0.57	1	0.45
Multicomponent	0.04	0.13	0.32	0.77	2.21	3	0.53
**BY OUTCOME**							
Evaluative WB	0.46	0.18	2.53	0.02	95.93	14	<0.01
Hedonic WB	0.35	0.16	2.22	0.04	236.68	26	<0.01
Eudemonic WB	0.58	0.13	4.47	0.047	2.80	2	0.25
QOL	0.77	0.25	3.07	0.02	44.76	8	<0.01

*SWB, subjective well-being; CB, cognitive behavioral; WB, well-being; SMD, standardized mean difference; SE, standard error.*

* *The variation in SMD attributable to heterogeneity was tested by Q statistic.*

We conducted a subgroup analysis by interventions grouped into physical activity (12 SMDs from six studies), mindfulness (13 SMDs from six studies), CB-based approach (11 SMDs from eight studies), and other psychological (12 SMDs from six studies), environmental (2 SMDs from two studies), and multicomponent interventions (4 SMDs from two studies). The pooled effects of mindfulness, CB-based approach, and other psychological interventions were significantly positive (*p* < 0.05) (Table 3). The effects of physical activity, environmental and multicomponent interventions were not significant (*p* = 0.10, 0.41, and 0.77, respectively) (Table 3).

Next, a subgroup analysis was also conducted by the SWB outcomes grouped into evaluative well-being (15 SMDs from 12 studies), hedonic well-being (27 SMDs from 18 studies), eudemonic well-being (3 SMDs from 3 studies), and mental component of QOL (9 SMDs from 7 studies). The pooled effects of interventions on every group of SWB outcomes (evaluative, hedonic, and eudemonic well-being, and the mental component of QOL) were significant and positive (*p* < 0.05) (Table 3).

## Discussion

### Summary of Evidence

The current study aimed to review systematically and conduct a meta-analysis of RCTs to improve SWB (evaluative, hedonic, and eudemonic well-being, and the mental component of QOL) of the working population. Among 39 included studies, physical activity (seven studies) and psychological interventions (e.g., mindfulness; six studies), CB-based approaches (eight studies), or other psychological interventions (seven studies) were most reported. Furthermore, ergonomics (three studies), environmental (three studies), multicomponent (two studies), and other interventions (three studies) were also included. From the results of the meta-analysis, the pooled effect of interventions on SWB was significantly positive; in addition, the effects of mindfulness, CB-based approach, and other psychological interventions were also significantly positive. These results could support the effectiveness of psychological interventions (e.g., mindfulness, CB-based approach, and other psychological interventions) for increasing SWB.

### The Effective Interventions for Improving Each Aspect of SWB

Among all included studies, half of them measured evaluative (18 studies) or hedonic well-being (19 studies). On the other hand, studies assessing eudemonic well-being (four studies) and the mental component of QOL (eight studies) were fewer. Thus, there is lack of evidence of intervention in increasing these two outcomes.

Among 21 studies assessing evaluative well-being, 10 reported significant improvement. Of significantly effective interventions, mindfulness (Hülsheger et al., 2013; Shonin et al., 2014; Crain et al., 2017) and environmental interventions (Linzer et al., 2015; Alhassan et al., 2016) were reported mainly. Mindfulness facilitates adaptive stress appraisal, which could help employees feel challenging work events as less stressful (Hülsheger et al., 2013; Crain et al., 2017), which may lead to a more positive evaluative judgment of one's work situation (e.g., job satisfaction) (Hülsheger et al., 2013). Additionally, environmental intervention could also be effective in improving job satisfaction (Linzer et al., 2015; Alhassan et al., 2016). For example, Linzer et al. (2015) reported improving communication and workflow were related to increasing job satisfaction. These favorable environmental factors (e.g., communication or workflow) were reported to be positively associated with job satisfaction by previous observational studies (Zangaro and Soeken, 2007; Linzer et al., 2009; Lu et al., 2019). Thus, improving these factors could enhance worker satisfaction (Linzer et al., 2015).

Nineteen studies measured hedonic well-being, by using the scale of happiness, emotional or mental well-being, vigor/vitality, work engagement, or positive affect. Thus, there is a variety of outcome measures of hedonic well-being. Among these, eight studies showed improvement of outcomes. For example, mindfulness could be effective (Aikens et al., 2014; Allexandre et al., 2016). Mindfulness consists of developing focused attention, nonjudgmental awareness, openness, curiosity, and acceptance of internal and external present experiences, all of which would help individuals act more reflectively (Allexandre et al., 2016). Recently, mindfulness has been adopted as an approach for decreasing emotional distress and maladaptive behavior (Bishop, 2004; Aikens et al., 2014). Therefore, mindfulness could also enhance hedonic well-being effectively. It is consistent with Knight et al. (2019), which reported mindfulness could be useful in increasing hedonic well-being (e.g., work engagement) (Knight et al., 2019).

Because the RCTs measuring eudemonic well-being were few, further RCTs aiming to increase it should be conducted. However, among four included studies, three were useful for increasing eudemonic well-being: psychological interventions such as CBT (Bolier et al., 2014), resiliency training (Waite and Richardson, 2004), and physical activity such as yoga (Hartfiel et al., 2011). Considering yoga contains psychological components (e.g., mindfulness) (Hartfiel et al., 2011), these effective interventions used some kind of psychological strategies (Waite and Richardson, 2004; Hartfiel et al., 2011; Bolier et al., 2014). This result corresponded with that of the general population (Weiss et al., 2016). In order to improve eudemonic well-being, people may need to review and judge the meaning and purpose of life (Steptoe et al., 2015), which could be facilitated by psychological intervention.

According to the mental component of QOL, some studies suggested that physical activity (e.g., coordination and flexibility exercise) (Brand et al., 2006), aerobic and weight-training (Atlantis et al., 2004), and psychological intervention (e.g., mindfulness) (Allexandre et al., 2016) could be effective.

The meta-analysis found a significantly positive effect of interventions on overall SWB. Specifically, the subgroup analyses showed that the effects of psychological interventions (e.g., mindfulness, CB-based approach, and other psychological interventions) on overall SWB were significantly positive (Table 3). Thus, these types of interventions could improve SWB effectively. On the other hand, the effects of environmental and multicomponent interventions were insignificant (Table 3), possibly due to the lack of statistical power because the number of studies included in the analysis was only two and four, respectively.

Furthermore, the effects of interventions on all aspects of SWB (evaluative, hedonic, and eudemonic well-being, and the mental component of QOL) were also significantly positive (Table 3). This result could indicate that all aspects of SWB could be enhanced by interventions.

### Practical Implication

The current systematic review and meta-analysis could indicate that psychological interventions (e.g., mindfulness, CB-based approach, and other psychological interventions) were effective in increasing SWB among working populations. Thus, for promoting workers' SWB, these strategies could be useful. For example, psychological interventions such as mindfulness (Hülsheger et al., 2013; Aikens et al., 2014; Shonin et al., 2014; Allexandre et al., 2016; Crain et al., 2017), CBT (Bolier et al., 2014) and resiliency training (Waite and Richardson, 2004) could increase SWB effectively.

### Risk of Bias Within Studies Applying Psychosocial Intervention

Based on our assessment of risk of bias, most of the included studies had high risk of blinding of participants and personnel, providers, and outcome assessments. For intervention study approaching workplace psychosocial factors (e.g., group-based workshop, and individual counseling) in the research field of social medicine, it may be difficult to conduct blinding of participants and providers. Furthermore, because most of the studies used self-administered questionnaires, blinding of outcome assessment may also be impossible. For further study using psychosocial intervention, these risks of bias should be reduced, for example, by using structured interview assessment.

### Limitations

The present systematic review has several limitations. First, this review is limited by English language restriction; thus, studies in other languages may have been missed. Second, there may be additional studies, especially those with negative results, that have been performed but not published. We assessed publication bias by drawing a funnel plot and conducting Egger's test, which was significant. Thus, sensitive analyses for studies reporting relevant SMDs or their SEs were also conducted, showing a significantly positive pooled effect of interventions on SWB.

## Conclusions

The current study revealed the effectiveness of interventions for increasing SWB. Especially, psychological interventions (e.g., mindfulness, CB-based approach, and other psychological interventions) may be useful to improve SWB.

## Author Contributions

ASa, KI, KW, YA, EA, HE, NN, YK, HA, and MI contributed to shifting, full text review, and extraction of information from each of the included studies for systematic review. ASa, KI, KW, and NS collected information from each study for meta-analysis. ASa, KI, KW, HE, AI, and NS independently assessed the included study quality using the risk of bias assessment tool. All authors conceived of the study, developed the study design, prepared the first draft, and approved the final manuscript.

## Conflict of Interest

The authors declare that the research was conducted in the absence of any commercial or financial relationships that could be construed as a potential conflict of interest.
