# Corrigendum: What kind of intervention is effective for improving subjective well-being among workers? A systematic review and meta-analysis of randomized controlled trials

**DOI:** 10.3389/fpsyg.2023.1236746

**Published:** 2023-07-03

**Authors:** Asuka Sakuraya, Kotaro Imamura, Kazuhiro Watanabe, Yumi Asai, Emiko Ando, Hisashi Eguchi, Norimitsu Nishida, Yuka Kobayashi, Hideaki Arima, Mai Iwanaga, Yasumasa Otsuka, Natsu Sasaki, Akiomi Inoue, Reiko Inoue, Kanami Tsuno, Ayako Hino, Akihito Shimazu, Akizumi Tsutsumi, Norito Kawakami

**Affiliations:** ^1^Department of Public Health, School of Medicine, Tokyo Women's Medical University, Tokyo, Japan; ^2^Department of Mental Health, Graduate School of Medicine, The University of Tokyo, Tokyo, Japan; ^3^Center for Cancer Control and Information Services, National Cancer Center, Tokyo, Japan; ^4^Department of Public Health, Kitasato University School of Medicine, Kanagawa, Japan; ^5^Kyoto Industrial Health Association, Kyoto, Japan; ^6^Department of Psychiatric Nursing, Graduate School of Medicine, The University of Tokyo, Tokyo, Japan; ^7^Faculty of Human Sciences, University of Tsukuba, Tokyo, Japan; ^8^School of Health Innovation, Kanagawa University of Human Services, Kanagawa, Japan; ^9^Department of Mental Health, Institute of Industrial Ecological Sciences, University of Occupational and Environmental Health Japan, Fukuoka, Japan; ^10^Faculty of Policy Management, Keio University, Kanagawa, Japan

**Keywords:** subjective well-being, positive mental health, systematic review, intervention, worker, meta-analysis

In the published article, there was an error in affiliation 3 as published. The affiliation was listed as “Center for Cancer Control and Information Services, National Cancer Center Japan, Tokyo, Japan” but should be “Center for Cancer Control and Information Services, National Cancer Center, Tokyo, Japan.” Affiliation 3 has now been updated.

In the published article, there was an error. In the results section, “one meditation awareness training (acceptance commitment therapy: ACT)” was written in error. The correct term is “one meditation awareness training (MAT) intervention.”

A correction has been made to *Results, Mindfulness*. The corrected section is shown below.

Six mindfulness intervention studies were included. Among these, three were mindfulness-related group sessions (Aikens et al., 2014; Van Berkel et al., 2014; Crain et al., 2017), one was self-training (Hülsheger et al., 2013), and the other was a web-based program (Allexandre et al., 2016). In addition, one meditation awareness training (MAT) intervention was also reported (Shonin et al., 2014). These mindfulness programs were effective for improving evaluative well-being (e.g., job satisfaction and life satisfaction) (Hülsheger et al., 2013; Shonin et al., 2014; Crain et al., 2017), hedonic well-being (e.g., vigor/vitality) (Aikens et al., 2014; Allexandre et al., 2016), and the mental component of QOL (Allexandre et al., 2016).

There was also an error in Table 1 as published. The “period” and “number and hours of session” of the intervention in column “Core intervention component” for Bolier et al. (2014) were displayed as “6 weeks−12months” and “4–8 weekly sessions”, respectively. The correct terms are “4 weeks−5months” and “a few sessions or modules,” respectively. The corrected Table 1 and its caption appear below.

**Table 1:** The characteristics of the studies included in the systematic reviews (*N* = 39).

**Author/Year**	**Country**	**Population**	**Gender Number (%) of men**	**Age Mean (SD)**	**Core intervention component**	**SWB outcomes (scale if applicable)**	**Result on SWB outcomes**
Physical activity							
Puig-Ribera et al., 2008	Spain	Employees of university	All: 21 (30.0%)	Not listed	Walking program Period: 9 weeks Number and hours of session: not listed Instrument: pedometer and a map with some examples of walks Provider: not listed	Mental health (SF-12): Mental component of QOL Vitality (SF-12): Hedonic	Mental health: 0 Vitality: 0
Mansi et al., 2015	New Zealand	Employees of large meat processing plant	Int: 40 (12.2%) Cont: 14 (48.4%)	Int: 43 (14.9) Cont: 40 (12.2)	Walking program Period: 12 weeks Number and hours of session: not listed Instrument: physical activity booklet Provider: psychotherapist	Mental health (SF-36): Mental component of QOL	Mental health: 0
Sjogren et al., 2006	Finland	Employees of the city of Kuopio central administration	All: 24 (26.7%)	All: 45.7 (8.6)	Light resistance training, guidance on postural and movement control Period: 15 weeks Number and hours of session: not listed Instrument: resistance equipment Provider: psychotherapist	Life satisfaction (the scale from Ojanen, 1994 and 2000): Evaluative Meaning of life (the scale from Ojanen, 1994 and 2000): Eudemonic	Life satisfaction: 0 Meaning of life: 0
Brand et al., 2006	Germany	Employees (office and blue color workers)	Int: 36 (69.2%) Cont: 47 (90.4%)	Percent per age groups; age 20–35, 10.9%; age 36–45, 50.0 %; age 46–55, 28.2 %; age 56–65, 10.9%	Muscular relaxation, strengthening, coordination and flexibility exercises Period: 13 weeks Number and hours of session: not listed Instrument: not listed Provider: fitness coach	Psychological domain of quality of life (the World Health Organization Quality of Life inventory): Mental component of QOL Job satisfaction (the Life Satisfaction Questionnaire): Evaluative	Psychological domain of quality of life: + job satisfaction: 0
Hartfiel et al., 2011	England	University employees	Int: 3 (15.0%) Cont: 1 (5.0%)	Int: 40.6 (11.4) Cont: 38 (9.58)	Dru yoga intervention Period: 6 weeks Number and hours of session: 60 min class per week Instrument: CD and home practice form Provider: certified instructor	Life purpose and satisfaction (the Inventory of Positive Psychological Attitudes): Eudemonic	Life purpose and satisfaction: +
Atlantis et al., 2004	Australia	Star City casino employees	Int: 9 (45.0%) Cont: 11 (45.8%)	Int: 30 (6.8) Cont: 33 (8.3)	Aerobic and weight-training exercise Period: 24 weeks Number and hours of session: not listed Instrument: personalized e-mail Provider: not listed	Vitality (SF-36): Hedonic Mental health (SF-36): Mental component of QOL	Vitality: + Mental health: +
Strijk et al., 2013	Netherlands	Employees from academic hospital	Int: 93 (25.3%) Cont: 86 (23.7%)	Int: 52.5 (4.8) Cont: 52.3 (4.9)	A vitality exercise program (VEP); providing free fruit, personal coaching, yoga group, and aerobic session Period: 6 months Number and hours of session: not listed Instrument: not listed Provider: qualified instructor	Vitality (RAND-36 vitality scale): Hedonic Vitality (UWES): Hedonic	Vitality (RAND-36 vitality scale): 0 Vitality (UWES): 0
Ergonomics							
Figl-Hertlein et al., 2014	Australia	Teachers of secondary school	Not listed	Not listed	Ergonomics individual training (exercise and functional training), and stress management training Period: 5 months Number and hours of session: 2 sessions (3–4 h) Instrument: not listed Provider: licensed psychotherapist	Mental health (SF-36): Mental component of QOL Emotional well-being (AVEM): Hedonic	Mental health: 0 Emotional well-being: 0
Haukka et al., 2010	Finland	Kitchens of schools, kindergartens and nursing homes	Int: 167 (63.5%)Cont: 143 (59.3%)	Int: Range = 19–63 Median = 46 Cont: Range = 19–62 Median = 47	Ergonomics participatory training Period: approximately 11–14 months Number and hours of session: 8 sessions (total 28 h, each 3–5 h) Instrument: not listed Provider: researcher	Job satisfaction (“How satisfied are you with your present work?”): Evaluative	Job satisfaction: –
King et al., 1997	Not listed	Employees of manufacturing industry	Not listed	Not listed	Ergonomics participatory training with job redesign Period: 2–5 weeks Number and hours of session: 2 sessions (1 h) Instrument: not listed Provider: researcher, occupational therapist and safety professional	Job satisfaction (The Minnesota Satisfaction Questionnaire): Evaluative	Job satisfaction: +
Psychology (mindfulness)							
Hülsheger et al., 2013	Germany	Employees in hospitals, schools, kindergartens, and medical practices	All: 18 (28.1%)	All: 38.6 (11.1)	Mindfulness-based cognitive therapy and mindfulness-based stress reduction (MBSR), self-training Period: 2 weeks (10 working days) Number and hours of session: no session Instrument: diary booklet, a CD, postcard, and daily e-mail Provider: not listed	Job satisfaction [five items from Judge, Locke, Durham, and Kluger (1998)]: Evaluative	Job satisfaction: +
Crain et al., 2017	Canada and United States	Teacher	11% Number of men is not listed	46.9 (9.2)	Mindfulness training program based on MBSR, group session and homework Period: 8 weeks Number and hours of session: 11 group sessions (2–7 h/sessions, total 36 h) Instrument: not listed Provider: instructors having formal professional training of MBSR	Satisfaction with work life (“Overall, how satisfied are you with your present teaching job?”): Evaluative Satisfaction with home life (“Overall, how satisfied are you with your life at home?”): Evaluative	Job satisfaction: + Life satisfaction: +
Van Berkel et al., 2014	Netherlands	Employees from Dutch research	Int: 36.4% Cont: 28.9% Number of men is not listed	Int: 46.0 (9.4) Cont: 45.1 (9.6)	Mindfulness-based training, free , lunch walking, and buddy-system, group session and e-coaching Period: 6 months Number and hours of session: 8 weekly group sessions (90 min) Instrument: e-coaching, CD, booklet Provider: certificated trainer	Work engagement (UWES): Hedonic	Work engagement: 0
Aikens et al., 2014	Michigan	Dow employees	Not listed	Range = 18–65	Mindfulness program, group session and individual online training Period: 7 weeks Number and hours of session: 7 times weekly (1 h) Instrument: web site and workbook Provider: certified medicine physician with MBSR training	Vigor (Shirom Vigor Scale): Hedonic	Vigor: +
Allexandre et al., 2016	United States	Employees of call center	All: 16.8% Number of men is not listed	All: 40.0 (12.6)	Online mindfulness stress management program (WSM), with weekly group meeting (WSMg1), with weekly group meeting and expert clinical support (WSMg2) Period: 8 weeks Number and hours of session: 8 weekly group sessions (1 h) Instrument: online program, CD, and diary article Provider: licensed clinical counselor and social worker	Mental health (SF-36): Mental component of QOL Vitality (SF-36): Hedonic	Mental health: + (WSM and WSMg1) Vitality: + (WSM and WSMg1)
Shonin et al., 2014	United Kingdom	Employees with middle management responsibility	Int: 56.9% Cont: 56.9% Number of men is not listed	Int: 40.14 (8.11) Cont: 39.91 (8.67)	Meditation Awareness Training (MAT), group and individual session Period: 8 weeks Number and hours of session: 8 weekly group sessions (90 min), and 4 weekly individual sessions (50 min) Instrument: CD Provider: researcher having psychotherapy and meditation teaching experience	Job satisfaction (Abridged Job in General Scale): Evaluative	Job satisfaction: +
Psychology (CB based approach: CBT)							
Umanodan et al., 2014	Japan	Employees in a manufacturing company	Int: 135 (95.1%) Cont: 109 (90.1%)	Int: 39.7 Cont: 38.0 SD is not listed	Computer based stress management (problem-solving, time management, assertion and delegation, cognitive reconstruction and causal attribution), individual training Period: 7.4 weeks Number and hours of session: 6 sessions Instrument: e-mail Provider: the author	Job satisfaction (BJSQ): Evaluative Work engagement (UWES-J): Hedonic	Job satisfaction: 0 Work engagement: 0
Bond and Bunce, 2000	Not listed	People in a large media organization	All: 15 (50.0 %)	36.43 (9.72)	Acceptance commitment therapy (ACT) training, group session Period: 3 months Number and hours of session: 3 sessions (3.25 h per session) Instrument: not listed Provider: not listed	Intrinsic job satisfaction (work and life attitude survey): Evaluative	Intrinsic job satisfaction: 0
Billings et al., 2008	United States	Employees in a major technology company	All: 91 (29.4%)	Percent per age groups; age 20–29, 24.4%; age 30–39, 51.1%; age 40–49, 20.2%; age 50–59, 3.3%; age 60–69, 1.0%	Online stress and mood management training (goal setting, problem solving, and cognitive reconstruction) Period: 3 months Number and hours of session: not listed Instrument: not listed Provider: not listed	Positive mood (the Positive and Negative Affect Schedule): Hedonic	Positive mood: 0
Psychology (CB based approach: CBT)							
Bolier et al., 2014	Netherlands	Nurses and allied health professionals	Int: 43 (22.9%) Cont: 31 (17.4%)	Int: 42 (11.4) Cont: 38 (12.1)	Tailored online interventions based on CBT Period: about 4 weeks−5 months Number and hours of session: a few sessions or modules Instrument: web site and e-mail Provider: not listed	Positive mental health (The Mental Health Continuum — Short Form): Eudemonic	Positive mental health: +
Psychology (CB based approach: cognitive approach)							
Unsworth and Mason, 2012	Not listed	White-collar professional technical staff in the public sector	Int: 22 (57.9%) Cont: 20 (60.6%)	Int: 46.78 (range = 37–59) Cont: 44.65 (range = 24–58) SD in not listed	Online self-leadership training (self-management strategies and cognitive restructuring) Period: 10 weeks Number and hours of session: 5 modules (2 h), 1 module per 2 weeks Instrument: not listed Provider: an expert facilitator	Positive affect (Job Affect Scale): Hedonic	Positive affect: +
Sanders et al., 2011	Australia	Employees in various organization (having a child aged between 1 and 16 years)	The majority of parents participating in the study were mothers (72.4%)	Not listed	Workplace Triple P: training of work-family balance coping skills (e.g., cognitive reconstruction), and positive parenting skills, group sessions and telephone consultations Period: 8 weeks Number and hours of session: 4 times group session (2 h), and 4 individual telephone consultations (15 to 30 min) Instrument: workbook Provider: trained practitioner	Job satisfaction (Work and Life Attitude Scale): Evaluative	Job satisfaction: +
Psychology (CB based approach: behavioral approach)							
Vuori et al., 2012	Finland	Employees in human resources development departments and occupational health services	Int: 50 (13.6%) Cont: 36 (10.3%)	Int: 50.47 (6.49) Cont: 49.67 (6.44)	The enhancement of career management skills (e.g., communication and assertion), group session Period: 3–7 days Number and hours of session: 5 sessions (4 h), or over 3 full days Instrument: not listed Provider: trainer	Work engagement (UWES-9): Hedonic	Work engagement: 0
Barbosa et al., 2015	Portugal	Workers in aged care facilities	All participants were women	Int: 43.37 (10.00) Cont: 45.90 (8.04)	Person centered care (PCC) based psycho-educational (PE) intervention (e.g., time management and problem-solving), group session Period: 8 weeks Number and hours of session: 8 weekly sessions (90 min) Instrument: hand-outs Provider: psychotherapist	Job satisfaction (the short-form Minnesota Satisfaction Questionnaire): Evaluative	Job satisfaction: 0
Psychology (others)							
Waite and Richardson, 2004	United States	Managers and employees in large government organization	Int: 12 (16.4%) Cont: 12 (15.6%)	Percent per age groups; age 18–33, 59.3%; age 34–49, 28.0%; over 50; 12.0%	Resiliency training program, group session Period: 5 weeks Number and hours of session: 5 weekly sessions (7 h), and follow-up review session for managers were provided every other week (1–2 h) over 6 weeks Instrument: not listed Provider: trainer	Purpose in life (the Purpose in Life Test): Eudemonic Job satisfaction (the SURVEY2000 IRS/NTEU Employee Satisfaction instrument): Evaluative	Purpose in life: + Job Satisfaction: +
Fillion et al., 2009	Canada	Palliative care nurses	Int: 1.8% Cont: 0% Number of men is not listed	Int: 44.96 (9.61) Cont: 43.13 (11.56)	Meaning centered training; covering five principal themes of Viktor Frankl's logotherapy, group session Period: 4 weeks Number and hours of session: 4 weekly sessions Instrument: facilitator manual book Provider: facilitator licensed psychologist and received training	Job satisfaction (General Satisfaction subscale of the Job Diagnostic Survey): Evaluative The spiritual quality of life (the Spirituality subscale of the Functional Assessment of Chronic Illness Therapy): Mental component of QOL The emotional quality of life [the Vigor/Activity subscale of the Shortened Profile of Mood States (POMS-37)]: Hedonic	Job satisfaction: 0 The spiritual quality of life: 0 The emotional quality of life: 0
Morgan and Harris, 2015	England	Workers in a medium-sized, further education college (during a period of organizational downsizing)	22 (33.3%)	45.18 (8.33)	The work-related self-affirming implementation intention (WS-AII) Period: not listed (one time session) Number and hours of session: one time Instrument: not listed Provider: not listed	Job satisfaction (the 16-item job satisfaction scale): Evaluative	Job satisfaction: 0
Muller et al., 2016	Germany	Nurses in community hospital	Int: 5.6% Cont: 5.9% Number of men is not listed	Int: 44.67 (9.34) Cont: 42.74 (9.91)	Selection, Optimization, Compensation (SOC) training; training of coping with job demand or job resource, group session Period: 9 months Number and hours of session: 6 sessions (0.5–8 h, interval: 2–8 weeks) Instrument: manuals, worksheets, and diary Provider: trainer (experienced occupational health professional)	Mental well-being (WHO-5): Hedonic	Mental well-being: +
Feicht et al., 2013	Germany	Employees in local insurance company	Int: 13 (24.1%) Cont: 18 (38.3%)	Int: 37.61 (7.72) Cont: 36.77 (10.42)	Online happiness training (e.g., “How do you feel? Check your state of mind”) Period: 7 weeks Number and hours of session: 7 weekly sessions (10–15 min) Instrument: e-mail Provider: not listed	Happiness and satisfaction (Visual Analog Scale): Hedonic Mental well-being (WHO-5): Hedonic	Happiness and satisfaction: + Mental well-being: +
Tuckey and Scott, 2014	Australia	Fire-fighters after potentially traumatic events (PTE)	All: 61 (91%)	Not listed	Critical incident stress debriefing (CISD), group session Period: one session Number and hours of session: one session (90 min) Instrument: not listed Provider: trained and experienced mental health professionals and peer supporters	Quality of life (Quality of life enjoyment and satisfaction questionnaire-short form): Mental component of QOL	Quality of life: 0
Coffeng et al., 2014	Netherlands	Office employees of a financial service provider	Int: 73 (61.9%) Cont: 65 (61.3%)	Int: 43.6 (10.3) Cont: 40.7 (9.2)	The social environmental intervention consisted of group motivational interviewing (GMI) Period: 6 weeks Number and hours of session: 3 times (90 min) Instrument: not listed Provider: trained team leader	Work engagement (UWES): Hedonic	Work engagement: 0
Environment							
Linzer et al., 2015	New York	Primary care clinician	Int: 39 (46.9 %) Cont: 41 (49.4 %)	Int: 48.3 (8.9) Cont: 46.4 (9.4)	Each clinic chooses a variety of methods to improve work life (e.g., improving communication and workflow) Period: not listed Number and hours of session: not listed Instrument: not listed Provider: not listed	Job satisfaction (Physician job satisfaction scale): Evaluative	Job satisfaction: +
Alhassan et al., 2016	Ghana	Staffs in health facilities accredited by the National Health Insurance Authority (NHIA).	Int: 40% Cont: 30%	Int: 38.3 (14.4) Cont: 36.5 (13.4)	Systematic Community Engagement (SCE) Intervention, assessing and improving of health service quality (e.g., staff attitude) Period: about 1 year Number and hours of session: not decided (on a regular basis for one year) Instrument: not listed Provider: trained facilitator	Staff motivation (Staff were asked to rank their motivation levels on 19 workplace motivation proxies): Evaluative	Staff motivation: +
Stansfeld et al., 2015	Not listed	Employees and managers in National Health Service (NHS) Mental Health Trust	Int: 74 (26.2%) Cont: 10 (15.0%)	Aged over 50 Int: 21 (31%) Cont: 112 (40%)	E-learning program for managers based on the Health and Safety Executive (HSE) management standards for work-related stress, face to face session and support by telephone Period: 3 months Number and hours of session: 1–2 modules weekly Instrument: not listed Provider: trained facilitator	Employee well-being (the Warwick Edinburgh Mental Wellbeing Scale): Hedonic	Employee well-being: 0
Multi component							
Roussel et al., 2015	Not listed	Hospital employees with an increased risk for the development of low back pain	Int: 5 (16.1%) Cont: 7 (18.4%)	Int: 41.4 Cont: 40.4 SD in not listed	A multidisciplinary prevention program for low back pain (LBP): physical activity, ergonomics, and psychological training Period: 3 months Number and hours of session: 10 group sessions (1 h), and 5 individual sessions Instrument: not listed Provider: physiotherapists, dietician, and occupational therapists	Vitality (SF-36): Hedonic Mental health (SF-36): Mental component of QOL	Vitality: 0 Mental health: 0
Sforzo et al., 2012	New York	Employees in the company's New York City main branch where more than 11,000 were employed	44 (45.8%)	34.5 (7.48)	The multipoint educational intervention: physical activity and psychological (stress management) intervention Period: 12 weeks Number and hours of session: several times educational sessions and weekly text messages, and twice cafeteria tours Instrument: web site, the fitness facility, and discount for healthy meal choices in the cafeteria Provider: not listed	Life satisfaction (The five-item Satisfaction with Life Scale): Evaluative Job satisfaction (Michigan Organizational Assessment Questionnaire Job Satisfaction Subscale): Evaluative	Life satisfaction: 0 Job satisfaction: 0
Others							
Schrijnemaekers et al., 2003	Netherlands	Caregivers for elderly persons	Int: 7 (4.6%) Cont: 10 (7.2%)	Int: 35.2 (9.3) Cont: 37.7 (8.6)	Emotion-oriented care training for caregivers (e.g., learning non-verbal communication toward the resident), group session, homework Period: 8 months Number and hours of session: 2 clinical lessons (1 h), 6-day training program, and 3 supervision meetings Instrument: video Provider: the qualified and experienced teacher of a professional training organization	Job satisfaction of the professional caregivers (Maastricht Work Satisfaction Scale for Healthcare): Evaluative	Satisfaction with opportunities for self-actualization: + Satisfaction with head of the ward: + Satisfaction with quality of care: 0 Satisfaction with contact with colleagues: 0 Satisfaction with contact with residents: 0
Backman et al., 2011	United States	Low-wage workers of apparel manufacturers or food processors	Int: 135 (34.5%) Cont: 46 (33.6%)	Int: 32.6 (8.3) Cont: 33.9 (10.1)	Providing fresh fruit at workplace Period: 12 weeks Number and hours of session: 3 days a week Instrument: fruit delivery Provider: fruit delivery company	Job satisfaction (using 3 items, including workers' satisfaction with their jobs, supervisors/managers, and companies): Evaluative	Job satisfaction: 0
Bittman et al., 2003	United States	Employees in a non-profit continuing care retirement community	24 (21.4%)	45.3 SD in not listed	Recreational music making intervention (e.g., a mind-body wellness exercise, activity using shaker, and playing drum), group session Period: 6 weeks Number and hours of session: 6 sessions (1 h) Instrument: hand drums, sound shapes, auxiliary percussion instruments , and a clavi nova Provider: trained facilitator	Vigor/activity (POMS): Hedonic	Vigor/activity: +

+, favorable effect; –, unfavorable effect; 0, no effect; SWB, subjective well-being; Int, intervention; Cont, control.

The authors apologize for these errors and state that they do not change the scientific conclusions of the article in any way. The original article has been updated.
