# Supplementary material for: What Kind of Intervention Is Effective for Improving Subjective Well-Being Among Workers? A Systematic Review and Meta-Analysis of Randomized Controlled Trials
Source: Front Psychol. 2020 Nov 13;11:528656. doi: 10.3389/fpsyg.2020.528656 (PMC7691289; doi:10.3389/fpsyg.2020.528656)
Supplement: Supplementary file 1 [file Table_1.DOCX]

Appendix 1: The details of search terms

(worker* OR worksite* OR workplace* OR employ*) AND ("quality of life" OR ((mean* OR purpose) AND life) OR ((psychological or mental or emotional) and (wellbeing OR well-being)) OR satisfaction OR engagement OR happiness OR (positive AND (affect OR emotion)) OR (absence AND "negative affect")) AND (randomized controlled trial [pt] OR (randomized [tiab] AND controlled [tiab] AND trial [tiab]))
